# The Impact of Nanoparticles and Molecular Forms of TiO_2_ on the Rhizosphere of Plants in the Example of Common Wheat (*Triticum aestivum* L.)—Shifts in Microbiome Structure and Predicted Microbial Metabolic Functions

**DOI:** 10.3390/ijms26020685

**Published:** 2025-01-15

**Authors:** Sebastian Wojciech Przemieniecki, Marta Damszel, Olga Kosewska

**Affiliations:** Department of Entomology, Phytopathology and Molecular Diagnostics, University of Warmia and Mazury in Olsztyn, Prawocheńskiego 17, 10-720 Olsztyn, Poland; marta.damszel@uwm.edu.pl (M.D.); olga.kosewska@uwm.edu.pl (O.K.)

**Keywords:** titanium dioxide, titanium dioxide nanoparticles, *Triticum aestivum*, microbiota, mycobiota, rhizobiome metabolism

## Abstract

This study investigated the effects of various titanium nanoparticles (TiO_2_NPs) on the structure, function, and trophic levels of the wheat rhizobiome. In contrast to the typically toxic effects of small nanoparticles (~10 nm), this research focused on molecular TiO_2_ and larger nanoparticles, as follows: medium-sized (68 nm, NPs1) and large (>100 nm, NPs2). The results demonstrated significant yet diverse impacts of different TiO_2_ forms on the rhizosphere microbiota. Large TiO_2_NPs2 and molecular TiO_2_ adversely affected the bacteriobiome and mycobiome, leading to an increase in autotrophic microbial groups. In contrast, medium-sized TiO_2_NPs1 shifted the microbiome toward chemoheterotrophy, promoting plant growth-associated bacteria, fungal saprotrophs, and potential phytopathogens, suggesting a beneficial r-strategy within the rhizosphere. Other treatments induced oligotrophic conditions, resulting in a less flexible rhizobiome with diminished root associations but an increased abundance of *Trichoderma* spp. Structural modelling revealed that even minor changes in operational taxonomic units (OTUs) could significantly alter the microbiota’s metabolic potential. These findings highlight the importance of further research to optimize nanoparticle applications for sustainable agriculture.

## 1. Introduction

The development of nanotechnology has resulted in intense pressure on the environment, particularly through the increasing production and application of metal nanoparticles in industries such as pharmaceuticals, medicine, chemicals, automotive, and agriculture. These nanoparticles, including titanium dioxide (TiO_2_), are widely valued for their antimicrobial properties, which enable their use as pesticides in crop production. However, the environmental impact of these nanoparticles, especially in terms of waste and ecological interactions, remains a subject of ongoing study. Research has shown that the size and chemical properties of nanoparticles can lead to varying environmental effects, necessitating detailed characterization of these interactions [1,2,3,4].

Intensive crop production is accompanied by the widespread use of agrochemicals, which maximizes crop yield and quality while exerting negative environmental impacts. Major advances have been made in cultivation, fertilization, and protection technologies for food crops, but not for fodder crops. The production methods and fertilization strategies for fodder crops, including forage grasses, are still insufficiently developed. Conventional disease, pest, and weed control in fodder crop production is nearly impossible. Therefore, alternative management options are being sought to optimize yields and improve crop quality. Nanotechnology could support forage grass production, with novel nanomaterials such as TiO_2_ nanoparticles applied as biostimulants to reduce the use of fertilizers and crop protection products without compromising efficiency. These nanoparticles also hold potential for enhancing the microbiological and chemical quality of forage grasses [4].

Titanium dioxide nanoparticles (TiO_2_NPs) have demonstrated both stimulatory and inhibitory effects on microorganisms, including bacteria and fungi [2]. At the cellular level, these nanoparticles can induce toxicity depending on factors such as size, dose, charge, and exposure time. Smaller nanoparticles (~10 nm) are particularly problematic, as they can penetrate cell membranes, disrupt cellular functions, and reduce chlorophyll synthesis in plants. Additionally, TiO_2_NPs can generate reactive oxygen species (ROS), damage DNA, and disrupt key metabolic processes, as evidenced by laboratory studies on mammals [3,5,6,7].

In soil environments, TiO_2_NPs influence microbial communities and their functions. Concentrations of these nanoparticles can alter bacterial abundance and metabolism, impacting processes like denitrification, sulphur oxidation, and nitrogen cycling. Similarly, their interactions with soil fungi can result in shifts in fungal diversity, including changes in dominant taxa and reductions in phytopathogenic species. While some studies highlight their potential to promote mycorrhizal fungi and plant-beneficial microorganisms, others report contrasting outcomes, reflecting inconsistencies in current research [8,9,10]. For example, Asadishad et al. [11] found that low TiO_2_ nanoparticle concentrations did not affect soil enzyme activity, whereas higher doses (≥100 mg kg^−1^) were detrimental. Similarly, Moll et al. [8] observed that high doses (1 g kg^−1^) could enhance nitrogen fixation but impair ammonia oxidation and nitrification after prolonged exposure. These findings underscore the complexity of nanoparticle interactions, with factors like particle charge further modulating their effects on rhizosphere dynamics and nutrient delivery [12,13,14].

Despite extensive research, the effects of TiO_2_NPs on rhizosphere microbiota remain unclear due to conflicting results. While some nanoparticles exhibit biostimulatory effects on microorganisms and support mycorrhization processes, others disrupt microbial communities. Furthermore, most studies focus on small nanoparticles (10–50 nm), with limited data on larger aggregates (≥100 nm), which are more representative of environmental conditions. Larger nanoparticles, while potentially less toxic, remain understudied in terms of their effects on living organisms [15,16].

This study aims to bridge these knowledge gaps by investigating the effects of TiO_2_NPs with varying sizes, including larger aggregates, on soil microbiome structure and function. The research evaluates changes in microbial physiology, biochemical traits, and their implications for plant growth and pathogen suppression. By identifying nanoparticle characteristics that influence microbial interactions, this study seeks to support the safe and effective application of TiO_2_ in agriculture. The findings will contribute to understanding how TiO_2_NPs impact biogenic element cycles and agroecosystem stability, enabling the development of strategies for their sustainable use.

## 2. Results

### 2.1. Bacteriobiome Characteristics

**Figure 1:**
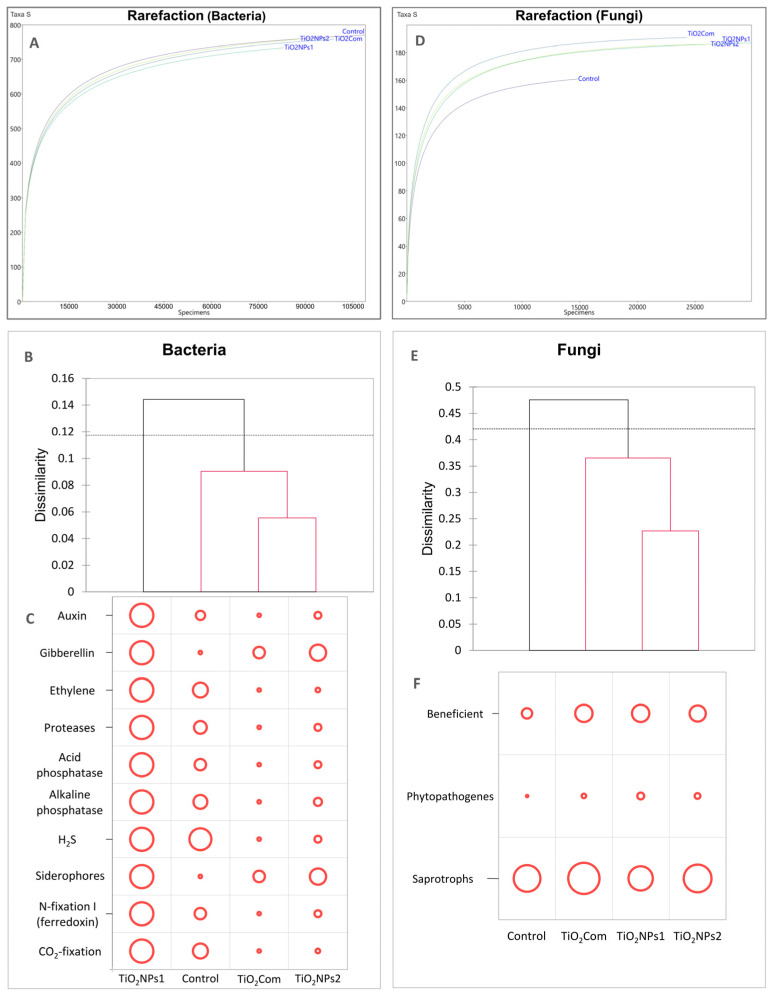
Rarefaction curves for bacterial and fungal communities (**A**,**D**), Bray–Curtis dissimilarity dendrograms (**B**,**E**) and the load of selected functional features of the bacteriobiome (**C**) and mycobiome (**F**).

Based on the results of the rarefaction analysis, it was observed that the differences in the abundance structures of individuals and the abundance of OTUs (operational taxonomic units) were similar in all treatments. The lowest abundances of individuals and OTUs were observed for TiO_2_NPs1, and the opposite results were obtained for the untreated rhizosphere (Figure 1A).

Based on the dissimilarity results based on the Bray–Curtis matrix for bacteriobiomes, a complete dissimilarity in the structure of OTUs for TiO_2_NPs1 was observed (branching above the threshold line). The remaining treatments did not show any significant differences from each other. However, the most remarkable similarity was observed between TiO_2_Com and TiO_2_NPs2. This group was the most different from the TiO_2_NPs1 treatment, while the control was characterized by partial similarity to both TiO_2_NPs1 and the TiO_2_Com-TiO_2_NPs2 group (Figure 1B).

In each treatment, the dominant bacterial OTUs included Vicinamibacterales (order), Gemmatimonadaceae (family), Vicinamibacteraceae (family), *Devosia* (genus), and Saprospiraceae (family). The use of TiO_2_NPs1 resulted in a significant reduction in the most numerous OTU, i.e., Vicinamibacterales (order). In the case of the remaining OTUs mentioned above, no significant changes were observed compared to the control. In the TiO_2_NPs1 treatment, an increase in the share of the following species was observed: *Arthrobacter*, *Polaromonas* and *Pseudomonas*, while after using both forms of the tested titanium dioxide nanoparticles, an increase in the share of *Acidibacter* spp. and the Rhizobiales order was observed. The commercial preparation did not affect the structure of the rhizosphere bacteriome. Regarding diversity indices, no clear differences were found between the treatments (Table 1).

In the case of the load carrying plant growth promotion (PGP) traits (Figure 1C), the total amount of loads reflected the dissimilarity presented at Figure 1B. The TiO_2_NPs1 treatment was characterized by the highest PGP potential for all 10 tested features. In control, the bacteriobiome had a high potential for H_2_S production and an average potential for producing auxins, ethylene, protease, phosphatases, N-fixation I, and CO_2_-fixation. A very low load of bacteria-producing gibberellins and siderophores was also observed in this treatment. TiO_2_Com and TiO_2_NPs2 treatments had similar feature loads. In both treatments, only an average potential for the production of gibberellin and siderophores was observed, and the remaining features were at a low level in the case of TiO_2_NPs2 or very low in the case of TiO_2_Com.

**Figure 2:**
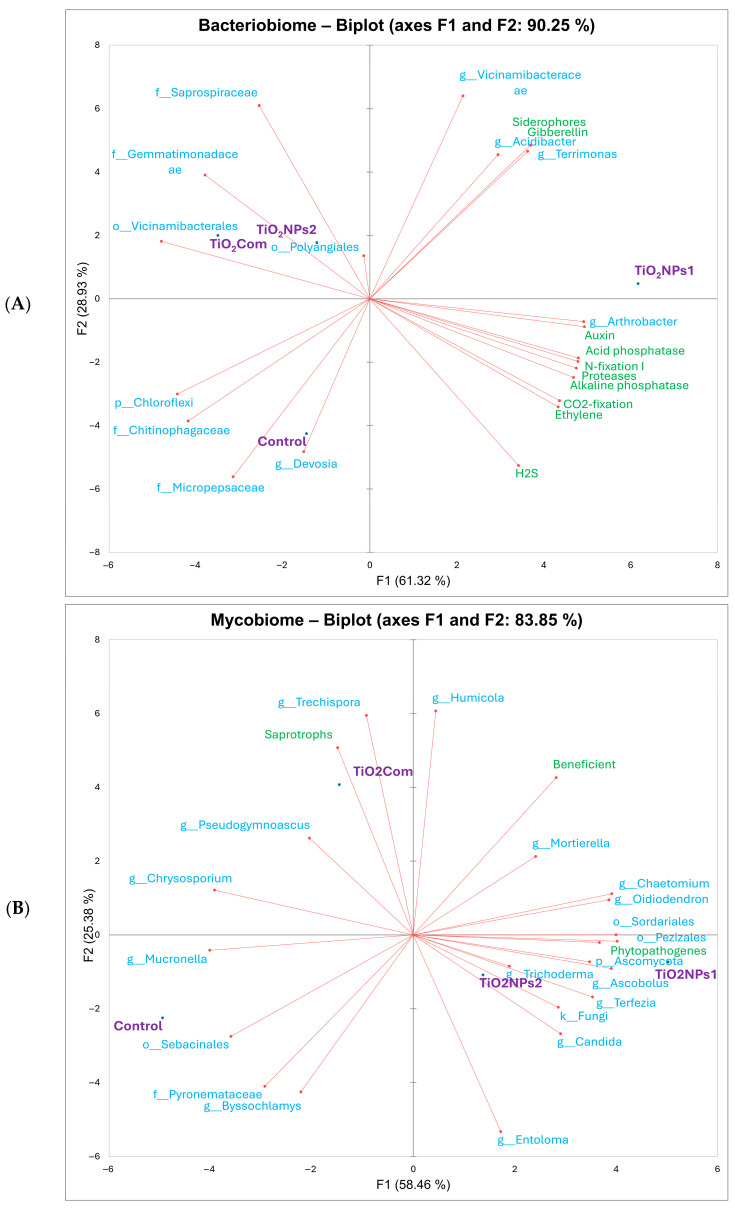
PCA showing overall relationships between bacterial OTUs and plant growth promotion traits (**A**) and fungal OTUs and trophic groups (**B**).

Based on the results of the PCA for bacteriobiomes and PGP traits (Figure 2), similar relationships between treatments were observed as in the dissimilarity analysis. The associated variables (high value traits) for the controls were Micropepsaceae, *Devosia*, Chitinophagaceae and *Chloroflexi*. TiO_2_Com-related variables included Vicinamibacteraceae, Gemmatimonadaceae and Saprospiraceae. In contrast, for TiO_2_NPs2, the same variables existed as for TiO_2_Com, in addition to the variables *Acidibacter* and *Terrimonas*, gibberellins and siderophores. For TiO_2_NPs1, these were all the PGP traits studied, plus *Acidibacter* and *Terrimonas*. Moreover, Vicinamibacteraceae were associated with all treated treatments. Note that most of the variables analyzed for the rhizosphere treated with any form of TiO_2_ were not associated with the control treatment (Figure 2A).

**Figure 3:**
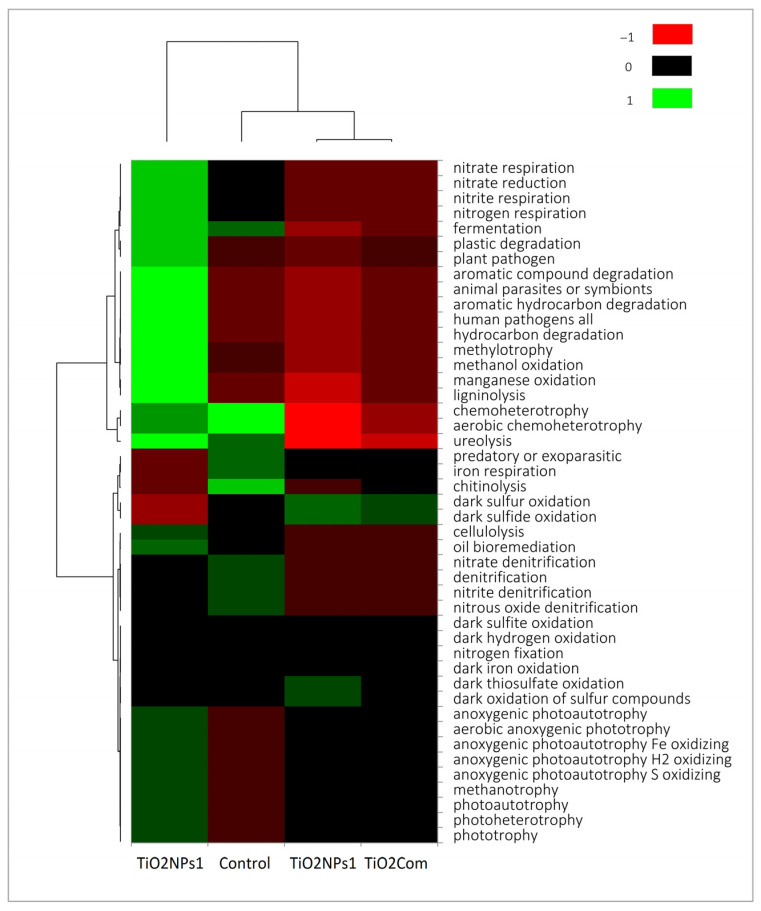
Heatmap of metabolic features for bacterial rhizobiome.

The TiO_2_NP1 treatment, unlike the other treatments, was characterized by the highest load of features indicating the chemoherotrophic nature of the bacteriobiome (N-cycle, decomposition of organic matter). However, compared to the control, it also had a higher load of photoautotrophs (Figure 3).

### 2.2. Mycobiome Characteristics

The results of the rarefaction analysis were different from those of the bacteriobiomes. The obtained curves revealed that each of the applied forms of TiO_2_ increased the size and density of OTUs compared to the control (Figure 1D).

Based on the dominance class analysis and Fisher’s exact test results, much greater differentiation was observed between OTUs of mycobiomes than between bacteriobiomes under the influence of the forms of titanium dioxide used. In the case of Sebacinales, a significant decrease in the share of this order of fungi was observed after the use of any of the tested forms of titanium dioxide. Moreover, in the case of TiO_2_NPs1, the reduction was significant (almost 6-fold), and this order passed from the class of eudominants to dominants. In the *Entoloma* genus case, a moderate share decrease (approximately 20%) was observed when TiO_2_Com was used. Phylum Ascomycota increased almost twice in the case of TiO_2_Com and significantly in the case of TiO_2_NPs1 and TiO_2_NPs2. With the addition of nanoparticles, the dominance class changed from subdominant to eudominant. In the case of the remaining OTUs, no eudomination was observed, but a large diversity of changes in OTU abundance was observed depending on the treatment with titanium dioxide forms. Moreover, a common feature of all forms was a moderate reduction in the share of Pyronemataceae and *Byssochlamys* OTUs (Table 2).

The treatment using the molecular form of titanium dioxide was characterized by the largest number of OTUs that moved from the occasional to the rare class. Nevertheless, a significant increase in the shares of *Humicola* and *Trechispora* was also observed, as well as their transition from the class of occasional to dominant individuals. Moreover, a moderate increase in the proportion of Chaetomium and Pseudogymnoascus was observed in TiO2Com. In the case of *Mortierella*, Sordariales, Pezizales, *Terfezia*, *Penicillium* and *Oidiodendron*, a moderate increase in these OTUs was observed after TiO_2_NPs1 treatment. In this treatment, a significant increase in the share of *Chaetomium* was also observed (over 13-fold) with a simultaneous change in the dominance class from occasional to subdominant individuals (Table 2).

Moreover, there was a significant decrease (22-fold) in the share of fungi of the *Chrysosporium* genus and a change in the dominance class from dominants to occasional individuals. The TiO_2_NPs2 treatment was characterized by a significant decrease in the share of *Chrysosporium* and a change in the class from dominant to rare individuals. A moderate increase in proportion and dominance class from occasional or rare to subdominant was observed for OTUs *Humicola*, *Chaetomium*, Sordariales, *Ascobolus*, *Pseudogymnoascus*, *Nadsonia*, *Terfezia* and *Trichoderma* (Table 2).

The calculated diversity indices for the mycobiome showed that the control was twice as dominant and had the lowest diversity and uniformity. The lowest dominance and the highest diversity characterized the TiO_2_Com and TiO_2_NPs1 treatments. Using each form of titanium dioxide resulted in a statistically significant change in the parameters of diversity indicators (Table 2).

In the case of Bray–Curtis dissimilarity, the structures of mycobiomes on which any form of titanium dioxide was used differed significantly from the control. TiO_2_NPs1 and TiO_2_NPs2 were the most similar to each other, while TiO_2_Com was an intermediate form between the control and mycobiomes on which nanoparticle forms were used (Figure 1E).

Based on a non-standardized analysis of general trophic features of mycobiomes, no clear changes in their share were observed. A slight increase in the share of potentially beneficial fungi was observed after adding each of the forms of TiO_2_ and a slight decrease in saprotrophs in favour of potential phytopathogens in the case of TiO_2_NPs1 (Figure 1F).

The PCA results (Figure 2) showed an analogous similarity to the dissimilarity results shown on the dendrogram (Figure 1E). Analyzing the associations between variables and treatments, it was observed that the variables with the highest values for the control were Pyronemataceae, Sebacinales, *Byssochlamys* and *Chrysosporium*. The variables correlated with TiO_2_Com included *Humicola*, *Trechispora*, and *Chrysosporium*, which were correlated with saprotrophs and beneficials. The TiO_2_NPs group was common to both treatments, although the correlations varied in strength. This group included *Entoloma*, Ascomycota, *Chaetomium*, *Ascobolus*, Pezizales, *Terfezia*, *Trichoderma*, and fungi OTUs, and weakly associated *Oidiodendron* and *Candida*. The large majority of these OTUs were correlated with phytopathogenes. However, all treatments except the control were associated with increased loads of fungal beneficiens (Figure 2B).

### 2.3. Predicting the Function of Microbiome, Network and PLS-PM Analysis

**Figure 4:**
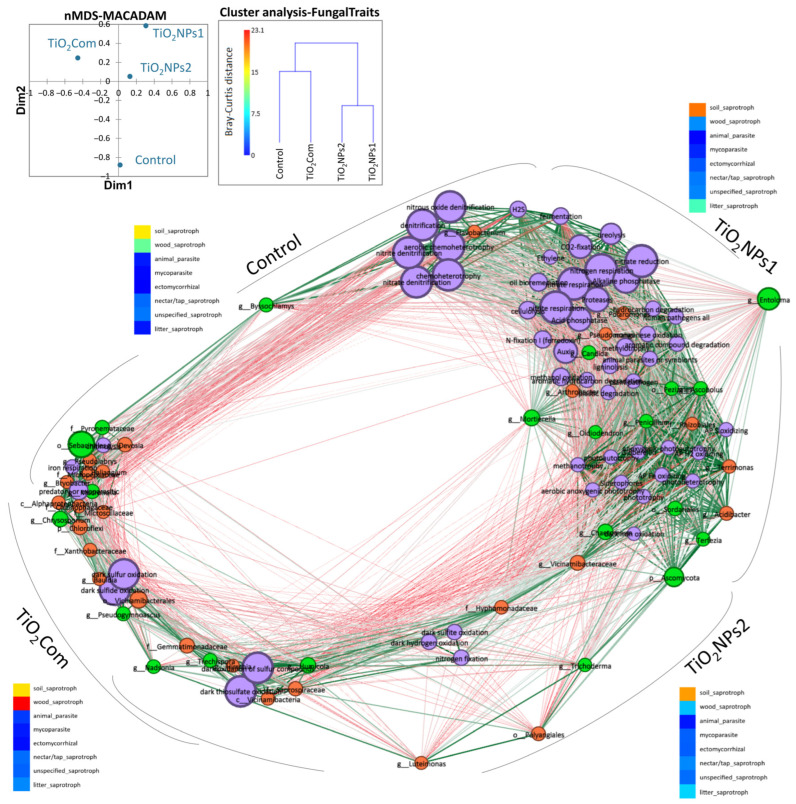
Network analysis of relationships between observed properties: microbiome, PGP-multitrais and physiological properties of bacteriobiome; the size of circles corresponds to the importance of the given feature in the analysis, the colour of lines shows correlations between properties (dark green, r = 1; grey, r = 0.75; dark red, r = 0.5; no links, r < 0.5). Colour of circles: orange—bacteriobiome OTUs; light green—mycobiome OTUs; violet—metabolic properties; blue (high correlation 1–0.75); and red (low correlation 0.75–0.5). The approximate link between the variant and the features is based on the location of the circle calculated on the Spearman correlation matrix. NMDS shows the distance between microbial metabolic properties, and cluster analysis shows dissimilarity between fungal trophic features.

Based on the analysis of potential physiological activity, it was observed that the overall highest activity occurred in the TiO_2_NPs1 treatment. It was also the treatment with the most significant difference compared to the other treatments, especially with regard to TiO_2_Com and TiO_2_NPs2. The highest number of microorganisms capable of the metabolism of a wide spectrum of organic substances and heterotrophic lifestyle, photoautotrophs and nitrogen respiration characterized the TiO_2_NPs1 treatment. The control was characterized by a high number of parasitic, chitinolytic and iron-respiratory bacteria. TiO_2_NPs1 and controls were collectively characterized by a microbiome capable of fermentation, chemoheterotrophy, and ureolysis. Dark sulphur/sulphide oxidation abundance was observed in the TiO_2_Com and TiO_2_NPs2 treatments. Nevertheless, the abundance of photoautotrophic bacteria was lower than in the TiO_2_NPs1 treatment but higher than in the control (Figure 3 and Figure 4).

More detailed data indicate that the TiO_2_NPs1 treatment showed the greatest number of associations between metabolic and microbial features. In this group, several key features of microbial metabolism were observed, such as those related to the reduction of nitrogen compounds, fermentation, proteolysis, ureolysis, cellulolysis, ligninolysis, CO_2_-fixation, production of phytohormones, acid and alkaline phosphatases, siderophore production, methylotrophy, sulphur and methanol oxidation, phototrophic processes, degradation of organic compounds, and, to a lesser extent, denitrification and chemoheterotrophy. These characteristics were closely associated with various OTUs of bacteria such as Pseudomonadaceae, *Arthrobacter*, *Polaromonas*, Rhizobiales, *Terrimonas*, *Acidibacter*, *Vicinamibacteraceae*, as well as with some OTUs of fungi such as *Entoloma*, *Mortierella*, *Candida*, *Chaetomium*, *Terfezia*, Sordariales, *Oidiodendron*, *Penicillium*, Pezizales, *Ascobolus* and Ascomycota (Figure 4).

The rhizosphere that was not treated with titanium dioxide compounds showed the formation of two subgroups that were not strongly related to each other. These two groups were linked by the fungus *Byssochlamys*, which was highly correlated with most traits in this group. The first subgroup included features related to denitrification, or aerobic or general chemoheterotrophy associated with *Flavobacterium*, H_2_S production and fermentation, and some parameters characteristic of the TiO_2_NPs1 treatment. The second group contained features partially shared with those of the TiO_2_Com treatment (titanium dioxide in the form of large nanoparticles). It included chitinolysis, iron respiration, and phytopathogenicity, with a high abundance of OTUs of the bacteria *Devosia*, *Pseudolabrys*, *Haliangium*, Micropepsaceae, *Briobacter*, Alphaproteobacteria, Chitynophagaceae, Microscillaceae, *Chloroflexi*, as well as OTUs of fungi Sebacinales, Pyronemataceae, *Mucronella* and *Chrysosporium* (Figure 4).

In the TiO_2_Com treatment, in addition to the subgroup containing features typical of the control rhizosphere, unique features were observed, such as oxidation of sulphur and sulphides in the dark, OTUs of Xanthobacteraceae, *Bauldia* and Vicinamibacterales bacteria, and *Pseudogymnoascus* and *Nadsonia* fungi. The third subgroup of the TiO_2_Com treatment contains features characteristic of the TiO_2_NPs2 treatment, such as sulphite and hydrogen oxidation in the dark, nitrogen fixation, and OTUs of the bacteria Hyphomonadaceae, *Luteimonas*, Polyangiales, Vicinamibacteraceae, as well as OTUs of the fungi *Trichoderma* and Ascomycota (Figure 4).

Results of analysis based on the FungalTraits database show that, under the influence of the forms of titanium used, there were changes in the number of groups such as soil saprotrophs, wood saprotrophs and letter saprotrophs. Changes in the abundance of groups such as animal parasites, mycoparasites, ectomycorrhizal, nectar/tap saprotrophs and unspecified saprotrophs were insignificant. The highest abundance of soil saprotrophs and wood saprotrophs co-dominances was observed in the control treatment. In the TiO_2_Com treatment, wood saprotrophs dominated, and soil saprotrophs co-dominated. Applying TiO_2_NPs1 and TiO_2_NPs2 nanoparticles increased the share of soil saprotrophs in the communities.

Nevertheless, an increase in saprotroph letters was observed in the case of TiO_2_NPs1. Based on cluster analysis, creating two similarity groups for fungal functional groups was possible. The first group included control and TiO_2_Com, and the second included TiO_2_NPs1 and TiO_2_. Nevertheless, the Bray–Curtis dissimilarity for mycobiome features in the first group was significant and amounted to 15, which could be compared to the dissimilarity in the nanoparticle group, which was 8 (Figure 4).

**Figure 5:**
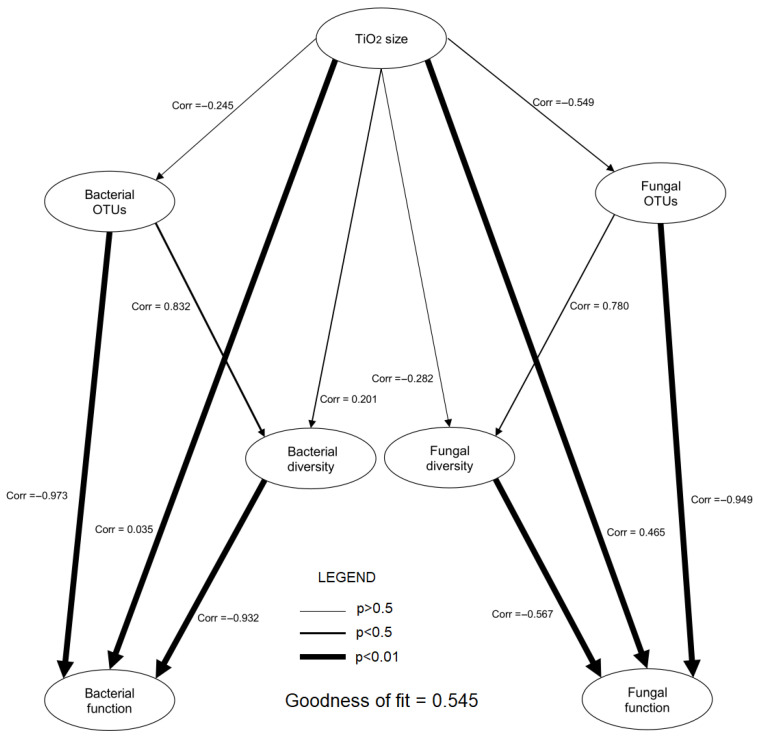
Partial least squares path models (PLS-PM) illustrating the direct and indirect effects of the interaction of size of TiO_2_ on bacterial and fungal rhizobiomes and their diversity and physiological properties.

In the partial least squares—path modelling (PLS-PM) analysis, the following correlation results between variables were obtained (*p* < 0.05): TiO_2_ size–Bacterial function r = 0.035, TiO_2_ size–Fungal function r = 0.465, Bacterial OTUs–Bacterial diversity r = 0.832, Bacterial OTUs–Bacterial function r = −0.973, Bacterial diversity–Bacterial function r = −0.932, Fungal diversity–Fungal function r = −0.567, and Fungal OTUs–Fungal function r = −0.949. No statistically significant correlation was observed between the remaining groups of variables. More detailed data are presented in Figure 5.

## 3. Discussion

### 3.1. Bacteriobiome

The structure of the bacterial rhizobiome did not undergo significant changes within individual OTUs. Nevertheless, regarding changes in dominance and abundance classes, the control was most similar to TiO_2_Com and least similar to nanoparticles. The TiO_2_NPs1 treatment showed the greatest difference, increasing the share of bacteria belonging to Actinobacteria and Proteobacteria. The taxa that increased their share in the community belonged mainly to heterotrophic microorganisms with high adaptability to environmental changes. However, together with heterotrophs, the number of photosynthetic microorganisms also increased. The greatest potential of PGP in this treatment is directly related to the presence of heterotrophs and the R strategy [17]. Despite large changes in the structure of OTUs between TiO_2_NPs1 and control, in the case of TiO_2_NPs1, the smallest changes in the functions represented by fungi were observed. The largest volume of features useful for plants represented by PGPR was characteristic of TiO_2_NPs1. However, a decline in other features was observed in TiO_2_Com and TiO_2_NPs2, apart from gibberellins and siderophores. The high PGP potential, represented by both the number of microorganisms and the number of features, indicates that rhizosphere bacteria can enter into symbiosis with plant roots, protecting them against pathogens. Such features are characteristic of heterotrophic bacteria and some photoautotrophs, enabling them to enter symbiosis with the plant. Indirectly, they also enable the survival of the rhizosphere structure in dynamically changing habitat conditions, such as unfavourable abiotic conditions or phytopathogen pressure. Moreover, with TiO_2_NPs1 treatment, a number of PGPR features that could potentially contribute to promoting plant growth and defending the plant against unfavourable biotic and abiotic factors were seen [17,18,19,20].

Among metal nanoparticles, the most attention is paid to silver, and there we observed certain regularities in relation to our results. Silver nanoparticles and their ionic forms are an excellent example of the impact of nanoparticles on the soil bacteriobiome. Research conducted in [21] proved that Ag nanoparticles significantly impact changes in coprophilic bacteria, especially beta-proteobacteria. However, the authors also emphasize that these changes result from interactions, so other environmental variables influence these changes, rather than adding various nanoparticle types.

Moll et al. [8] investigated the effect of higher doses of TiO2NPs than in this work. As a result, they obtained partially different results. The bacteriobiome was highly significantly modified under the influence of titanium dioxide nanoparticles. Moreover, Proteobacteria and *Cloroflexi*, but not Actinobacteria, were more likely to associate with very large nanoparticles (sizes > 100 nm). Proteobacteria, Firmicutes, Planctomycetes and some Actinobacteria were associated with large nanoparticles (145 nm), while most bacterial phyla but not Planctomycetes and *Chloroflexi* were associated with small nanoparticles (29 nm). However, the results of Moll et al. [8] partially differ from our results and indicate the effects of formation of the R strategy in small-sized TiO_2_-treated soil. However, it should be remembered that we examined the wheat rhizosphere in this study, not bulk soil.

### 3.2. Mycobiome

For the functional groups of mycobiomes analyzed, the nanoparticles were proven to interact with the rearrangement of the fungal community structure differently from the molecular form of titanium dioxide. Nevertheless, the moderate similarity of the TiO2Com mycobiomes concerning controls indicates that this form of titanium dioxide interacts with the structure of the mycobiomes in a unique way. A more detailed approach highlights that the main difference between the two groups (nanoparticle and control/ TiO_2_Com) is due to the fact that the application of both forms of nanoparticles drastically reduced the abundance of wood saprotrophs in favour of soil saprotrophs.

The most unique change for the particulate formed vis-à-vis the control was the significant increase in *Humicola* and *Trechispora* fungi. In the case of *Humicola* spp. the fungus was used to biotransform particulate forms of TiO_2_ to nanoparticulate forms that exhibit antibiotic activity [22]. This indicates that the genus of these fungi is insensitive to TiO_2_ nanoparticles, but also, during biotransformation, the particulate forms can participate in the intracellular metabolism of this organism. Therefore, it explains the more than 10-fold increase in the proportion of these fungi in the TiO_2_Com treatment and the predominant nature and noticeable increase in the proportion in the nanoparticle treatments. Fungi of the genus *Trechispora* are not described in the literature, which probably indicates the discovery of a strain or species resistant to TiO_2_ in particulate form.

Nevertheless, its deficient proportion in the other treatments may indicate the use of metal oxides in cellular metabolism. Moreover, the sensitivity of this genus to environmental metal pollution has been confirmed [23]. It should also be noted that the treatment caused an increase in the overall proportion of saprotrophs, which is mainly due to a significant increase in the proportion of the two fungal genera described above.

In the case of TiO_2_NPs1, a statistically significant increase in the proportion of fungal genera against the control was recorded only for *Chaetomium*. According to the FunglaTraits database, this fungus can be an endophyte of plants and has a hyper-fertile lifestyle. Nevertheless, under certain conditions, it causes soft rot. It can sometimes be a facultative plant pathogen, which is why, as in previous work, we also classified this fungal genus as a phytopathogen [3,24].

Both nanoparticle sizes used contributed to a significant reduction in the proportion of fungi of the genus *Chrysosporium*. As reported in the FungalTraits database, this is the fungus mainly responsible for wood decomposition, and, as such, may be responsible for the mineralization of organic matter in soil environments. A study by Binkauskiene et al. [25] used *C. merdarium* to investigate the degradation of TiO_2_ surfaces. The research team demonstrated that this species could grow and activate its metabolism when in contact with TiO_2_, but that successful survival was linked to the formation of 2,2,2-cryptand complexes with Ca^2+^ ions. This suggests that, for this species, the success of survival in the environment is related to the availability of calcium ions during exposure to TiO_2_. However, it needs to be confirmed whether this is the case for nanoparticle forms.

Concerning TiO_2_NPs1 and TiO_2_NPs2, it was observed that, although not significantly, a large number of OTUs moved two classes higher (from occasional to dominant individuals). A detailed discussion of each would reduce the readability of this research paper, so the focus is on describing global relationships in the following subsection. In contrast, this section focuses on a species of fungus with valuable, highly useful cultivated crops, i.e., *Trichoderma* sp.

It is difficult to explain the mechanism of resistance of Trichoderma fungi to low-particle TiO_2_ and its molecular forms. Nevertheless, the literature emphasizes that NPs of biological origin, including TiO_2_NPs of various types, can be synthesized using *Trichoderma* spp. This proves that the detoxification apparatus of this type of fungus is efficient, even to forms of metals with greater toxicity [26,27,28]. The above-mentioned observations explain why the *Trichoderma* genus was the most numerous in the “small TiO_2_” treatment. This proves that this fungus uses a vacant niche created after applying a form of Ti that is more toxic to the general rhizobiome community. It should be noted that fungi of the *Trichoderma* genus have a solid potential to resist and even absorb heavy metals from the environment. These fungi probably have metabolic mechanisms for tolerance or detoxification of titanium dioxide, unlike other fungi in the rhizobiome [29,30].

### 3.3. Relationship Between Changes in Microbiomes and Rhizobiome Metabolism

The results obtained using the network analysis module consolidated the knowledge indicating the comprehensive impact of various forms of titanium dioxide nanoparticles on the wheat rhizosphere microbiota and its potential consequences for the functioning of the rhizosphere ecosystem.

Concerning the rhizosphere fungal community, nanoparticles had a stronger effect than they had on bacteria. The changes in the trophic groups of fungi under the influence of the TiO_2_ forms used were not very drastic despite quite significant changes in the proportions and sizable changes in the dominance classes and taxonomic structures of mycobiomes. For bacteriobiomes, the opposite trend was observed. The insignificant changes in the abundance of many taxa were reflected in relatively drastic changes in the global metabolism of the bacteriobiomes.

Applying TiO_2_NPs2 nanoparticles increased the abundance of *Trichoderma* fungi in the rhizosphere fungal community. However, the high number of *Trichoderma* spp. was associated with a low number of bacterial functional groups, mainly chemoautotrophs. These results, combined with the ecological characteristics described by Ling et al. [17], indicate that *Trichoderma* requires special habitat conditions to thrive. Furthermore, it is likely that this fungus does not fully prefer dynamic and unstable bacterial rhizobiomes (r-strategy). Therefore, the genus grew best in the TiO_2_NPs2 treatment, which was moderately oligotrophic (strategy-K). For both nanoparticle types, there was a characteristic large group of unidentified Ascomycota, but also the highest proportion of typical soil saprotrophic fungi as seen in the heat map for the FungalTraits results at Network analysis. These fungi thrived best in the TiO_2_NPs1 treatment, as evidenced by the type of fungi readily decomposing organic matter, such as *Penicillium* sp., and possessing numerous traits helpful in maintaining the rhizosphere microbiome. Confirmed traits include the production of IAA, siferophores, or P-solubilization and, in the endophytic form, protection of the plant from the adverse effects of heavy metals, drought and cold [31]. In this case, bacteria in the TiO_2_NPs1 treatment also showed the potential described above, with an increased proportion of typical r-strategy bacteria belonging to Actinobacteria and Proteobacteria, indicating the intensification of heterotrophy. The structure of this rhizobiome brings the plant a complex of features that have a high potential for promoting growth, such as reduced nitrogen compounds, fermentation, proteolysis, ureolysis, cellulose and lignocellulose decomposition, CO_2_-fixation, production of phytohormones, acid and alkaline phosphatases, production of siderophores, methylotrophy, oxidation of sulphur and methanol, phototrophic processes and degradation of organic compounds. These features were correlated with different OTUs of bacteria and fungi and indicate the presence of an environmentally flexible rhizobiome, potentially beneficial for plant development [3,17,18,19,20]. The results of the PLS-PM analysis indicate significant relationships between microbial diversity and their ecosystem functions. For both bacteria and fungi, a negative correlation was observed between diversity (or the number of OTUs) and ecosystem function, suggesting that increased diversity does not always lead to improved ecosystem functions. The results suggest the possibility of a functional conflict, where a higher number of species may compete for resources, negatively impacting their functional capabilities, which corresponding with the previously mentioned strategies for the development of r and K. Additionally, the size of TiO_2_ particles has a clear impact on fungal functions, which may be important for further research into the effects of nanomaterials on microorganisms.

## 4. Materials and Methods

### 4.1. Experimental Setup

This study involved a pot experiment conducted with Bombona cultivar spring wheat, grown in soil with moderate organic matter content. The soil was amended with TiO_2_ nanoparticles of 68 nm (TiO_2_NPs1), 207 nm (TiO_2_NPs2), and a commercial TiO_2_ preparation. Each treated group received a final TiO_2_ concentration of 10 mg per kg of dry soil. Nanoparticles were purchased from Sigma Aldrich-MERCK Group (Darmstadt, Germany). Full property characterization and methodology were described in the previous work in the chapter “Physicochemical Characteristics of TiO_2_NPs” [4]. The control group contained untreated soil. The experiment ran until wheat reached the tillering stage, at which point rhizosphere samples were collected, and DNA was extracted from each sample. Detailed descriptions of the experimental design, including the cultivar, growth chamber conditions, pot setup, and sampling method, were previously documented by Gorczyca et al. [9], Przemieniecki et al. [32] and Przemieniecki et al. [3].

Soil for the experiment was sourced from the Agricultural Experimental Station in Bałcyny, Poland, known for a high prevalence of Fusarium infections. Samples were then transported to the University of Warmia and Mazury in Olsztyn. The experiment was established on the humus layer of Haplic Luvisol soil, classified as a silty sandy loam of moderate quality (class IVa) with the addition of 25% turf substrate to encourage the growth of eukaryotic organisms, per the WRB classification [33]. Soil was sieved to a 2 mm mesh, adjusted to 60% of its maximum water holding capacity, and placed into pots (2 kg per pot). Spring wheat (cv. Bombona) seeds were manually sown at a depth of 2 cm, with 6 seeds per pot. For additional control conditions, separate pots containing only soil without plants were prepared, to which either insect meal or nitrogen fertilizer was added at a rate of 180 kg N ha^−1^, alongside an unamended control. The experiment took place in a climate-controlled chamber for 30 days, with environmental settings maintained at 22 °C during the day and 18 °C at night, under a 12 h photoperiod, light intensity of 220 μmol photons m^−2^ s^−1^, and relative humidity of 80%. After germination, seedlings were thinned to 5 plants per pot as technical replicates. Following the plant growth period, the above-ground plant material and root systems were carefully removed from the pots, bulk soil was discarded, and the rhizosphere soil adhering to the roots was collected aseptically. Finally, roots were discarded, and rhizosphere samples were frozen at −20 °C for further analysis [3,9,32].

### 4.2. Sequencing

Genomic DNA was extracted from rhizosphere samples using the GeneMATRIX Soil DNA Purification Kit (Gdańsk, Poland). Prior to extraction, samples were homogenized with the TissueLyser LT (Qiagen, Hilden, Germany), using glass beads and the kit’s lysis buffer to ensure thorough mixing. Sequencing was carried out by Genomed (Warszawa, Poland), following a protocol outlined in earlier studies [34,35].

Microbial communities in the samples were analyzed by sequencing the V3–V4 region of the 16S rRNA gene for bacteria and the ITS region for fungi and other eukaryotes. Amplification of these gene regions was performed using Illumina-compatible primers: ITS3F (GCATCGATGAAGAACGCAGC) and ITS4R (TCCTCCGCTTATTGATATGC) for the fungal/eukaryotic ITS region, and 341F (CCTACGGGNGGCWGCAG) and 805R (GACTACHVGGGTATCTAATCC) for the bacterial 16S rRNA region. Illumina adapter overhang sequences were added to these primers for compatibility with the Illumina platform (San Diego, CA, USA). The amplicons were then indexed using the Nextera^®^ XT Index Kit (San Diego, CA, USA), following the manufacturer’s protocol, and sequenced on an Illumina MiSeq (San Diego, CA, USA), in paired-end mode (2 × 250 bp). The resulting sequencing data, saved in FASTQ format, were uploaded to the Metagenomics Rapid Annotation Subsystems Technology (MG-RAST) (https://www.mg-rast.org/ accessed on 10 April 2024) server for further analysis. Quality control steps included filtering out sequences with five or more ambiguous bases and those significantly deviating from the mean length (±2 standard deviations). Low-abundance sequences (singletons or those representing less than 0.0005% of total abundance) were removed from analysis. The sequences are accessible in the MG-RAST database under project ID mgp96669.

### 4.3. Statistical Calculation and Data Analysis

Taxonomic diversity of the analyzed OTUs was assessed using several indices: Chao1 for species richness, Simpson’s dominance index (λ) for measuring species dominance, Shannon’s diversity index (H’) for overall diversity, and Pielou’s evenness index (J’) to evaluate species distribution. Diversity indices and rarefaction curves were generated using PAST version 4.13 [36]. Dominance classes for bacterial and fungal communities were assigned based on previous studies by the authors of [37,38], respectively.

The MACADAM database [39] was utilized to infer microbial community functions, referencing the MetaCyc database for plant growth-promoting (PGP) properties [40] and FAPROTAX for metabolic function analysis [41]. Fungal trait analysis was conducted using the FungalTraits database [24].

Microbiome dissimilarities were calculated using Agglomerative Hierarchical Clustering (AHC) based on the Bray–Curtis method, with dendrograms created using Ward’s method. Pearson-based Mantel tests were used to compare the similarity matrices of different biome types. Principal Component Analysis (PCA) was conducted using a Pearson similarity matrix to analyze microbiome data (OTUs > 2%), PGP traits, and predicted microbial physiological characteristics. Heat maps and bubble charts were created to represent predicted metabolic functions and PGP characteristics sourced from the MACADAM and FungalTraits databases.

Partial least squares path modelling (PLS-PM) was applied to examine both direct and indirect effects of the studied variable groups. The model was built using standardized manifest variable weights, with centroid estimation applied for internal calculations. Correlations at a 0.001 significance level and model fit indices were calculated.

Statistical analyses were performed in XLSTAT [42] and PAST version 4.13 [36]. Network analysis was carried out using Gephi 0.9 with AxisForce 2 algorithm based on an “n-standardized” data matrix [43]. The statistical approach presented here was used in a previous study [3].

## 5. Conclusions

This study investigated the effects of titanium dioxide (TiO_2_) applied to soil in different particle forms—small-sized particles, medium-sized nanoparticles, and large nanoparticles—on the wheat rhizosphere microbiome and its functions. Our results indicate that TiO_2_ in its various forms significantly alters the taxonomic structure of the wheat rhizosphere microbiota, with implications for soil ecosystem processes, particularly biogeochemical cycles and microorganism interactions.

Notably, the application of large TiO_2_ nanoparticles (TiO_2_NPs2) negatively impacted both the bacteriome and mycobiome. Conversely, medium-sized nanoparticles (TiO_2_NPs1) were associated with a beneficial restructuring of the microbiome, fostering an environment conducive to plant growth by promoting a heterotrophic strategy within the rhizobiome. These findings highlight the potential of medium-sized nanoparticles (approximately 60 nm in diameter) to enhance the microbiome’s functional capacity, which could lead to improved plant health and productivity.

Our study also revealed that while TiO_2_ nanoparticle treatments altered the microbiome’s structure, these changes did not necessarily result in adverse effects on the biochemical potential of the rhizosphere. In fact, medium-sized nanoparticles showed the most favourable impacts, boosting microbial diversity and increasing the abundance of microorganisms that antagonize plant pathogens.

The comparison of different TiO_2_ forms demonstrated distinct patterns in the microbial community, with the smaller nanoparticles fostering a more flexible and diverse microbiome, while larger particles resulted in a more oligotrophic environment. These observations suggest that the application of TiO_2_ nanoparticles, particularly medium-sized ones, could play a role in promoting sustainable agricultural practises by enhancing microbial interactions in the rhizosphere.

In summary, the results of this study provide valuable insights into the influence of TiO_2_ nanoparticles on soil microbiomes and highlight their potential for improving crop production. Future research should focus on further elucidating the mechanisms underlying the interactions between nanoparticles and rhizosphere microorganisms, as well as their long-term impacts on soil health and ecosystem stability.

## Figures and Tables

**Table 1:** Structure of the bacteriobiome of the rhizosphere treated with different forms of TiO_2_.

OTU ID—BACTERIA	Control *	TiO_2_Com	TiO_2_NPs1	TiO_2_ NPs2
o__Vicinamibacterales	5.2	5.8	4.5	5.3
f__Gemmatimonadaceae	2.9	3.0	2.8	2.9
g__Vicinamibacteraceae	2.1	3.0	3.2	2.9
g__Devosia	2.5	2.2	2.2	2.4
p__Chloroflexi_KD4-96	2.1	1.8	1.3	1.6
f__Saprospiraceae	2.2	2.3	2.2	2.2
o__Polyangiales_BIrii41	1.7	1.5	1.6	2.1
g__Acidibacter	1.4	1.6	2.1	2.2
f__Xanthobacteraceae	1.4	1.4	1.2	1.4
g__Haliangium	1.3	1.1	1.0	1.1
f__Chitinophagaceae	1.5	1.1	0.5	1.1
f__Hyphomonadaceae	1.3	1.4	1.4	1.4
f__Micropepsaceae	1.4	1.0	0.7	1.0
c__Vicinamibacteria	1.2	1.4	1.2	1.2
c__Alphaproteobacteria	1.3	1.2	1.1	1.2
g__Bauldia	1.0	1.1	0.9	1.0
g__Terrimonas	1.0	1.2	1.5	1.4
g__Bryobacter	1.1	1.0	0.8	0.9
p__Chloroflexi_JG30	1.1	1.2	0.8	1.0
g__Pseudolabrys	1.1	0.7	0.5	0.8
g__Luteimonas	0.9	0.9	0.9	1.0
f__Rhizobiales	0.7	0.9	1.1	1.0
f__Microscillaceae	1.0	0.9	0.8	1.0
g__Hirschia	0.8	1.0	0.9	0.8
g__Arthrobacter	0.8	0.5	1.7	0.6
g__Flavobacterium	1.0	0.4	0.8	0.4
g__Polaromonas	0.6	0.4	1.2	0.5
g__Pseudomonas	0.3	0.1	1.2	0.2
OTUs	771	759	735	760
Simpson’s dominance (λ)	0.010	0.011	0.010	0.011
Shannon diversity (H′)	5.285	5.239	5.256	5.247
Pielou’s evenness (J′)	0.795	0.790	0.796	0.791

Colour intensity corresponds to dominance classes: dark—eudominant (>10%); medium—dominant (10–5%); light—subdominant (5–2%); faded—recedents (2–1%); white—subrecedents (<1%). OTU designations: k—kingdom; p—type; o—order; f—family; g—family; asterisks indicate the proportion of significantly different OTUs to from controls (Fisher’s exact test, *p* < 0.05.

**Table 2:** Structure of the rhizosphere mycobiome treated with different forms of TiO_2_.

OTU ID—FUNGI	Control	TiO_2_Com	TiO_2_NPs1	TiO_2_NPs2
o__Sebacinales	29.0	11.3 *	5.5 *	13.6 *
g__Entoloma	10.7	8.3	11.4	11.9
p__Ascomycota	3.0	5.6	10.0 *	11.5 *
g__Mortierella	3.2	4.3	5.5	2.8
g__Chrysosporium	6.8	5.5	0.3 *	1.9 *
g__Humicola	0.6	7.7 *	3.1	2.0
g__Chaetomium	0.4	3.1	5.4 *	4.3
o__Sordariales	0.7	1.9	3.8	3.0
f__Pyronemataceae	4.3	1.1	1.0	2.6
g__Mucronella	3.4	2.4	0.9	1.9
g__Byssochlamys	2.7	1.7	2.0	1.8
g__Candida	1.9	1.6	2.7	1.8
g__Ascobolus	0.3	1.0	4.4	2.1
g__Pseudogymnoascus	1.8	2.6	0.6	2.6
g__Trechispora	0.0	7.5 *	0.0	0.0
o__Pezizales	0.8	1.3	3.2	1.4
g__Nadsonia	1.3	1.7	1.0	2.3
g__Terfezia	0.0	0.4	2.6	2.8
g__Penicillium	0.9	1.3	2.0	1.5
g__Trichoderma	0.3	0.8	1.1	2.9
g__Oidiodendron	0.3	1.1	2.1	1.1
g__Phialemonium	0.8	1.8	0.1	0.7
k__Fungi	0.3	0.3	1.0	1.6
OTUs	161	191	187	186
Simpson’s dominance (λ)	0.111	0.045	0.043	0.057
Shannon diversity (H′)	3.224	3.750	3.743	3.618
Pielou’s evenness (J′)	0.634	0.714	0.715	0.692

Colour intensity corresponds to dominance classes: dark—eudominant (>10%); medium—dominant (10–5%); light—subdominant (5–2%); faded—recedents (2–1%); white—subdominant (<1%). OTU designations: k—kingdom; p—type; o—order; f—family; g—genus; asterisks indicate the proportion of significantly different OTUs to controls (Fisher’s exact test, *p* < 0.05).

## Data Availability

Data are available on request. Metabarcoding DNA sequence data for bacterial and fungal samples are available in the open access database: https://www.mg-rast.org/linkin.cgi?project=mgp96669 (accessed on 10 April 2024).
